# Hydrocortisone increases the risk of dysglycemia in critically ill patients

**DOI:** 10.1186/cc10764

**Published:** 2012-03-20

**Authors:** RT Van Hooijdonk, JM Binnekade, RE Harmsen, MJ Schultz

**Affiliations:** 1 Academic Medical Center, Amsterdam, the Netherlands

## Introduction

Hyperglycemia and hypoglycemia are independently associated with mortality and morbidity of critically ill patients [1,2]. Critically ill patients frequently receive hydrocortisone for refractory shock. While hydrocortisone infusion is associated with hyperglycemia [3], the effect of hydrocortisone on the incidence of hypoglycemia is uncertain. We hypothesized hydrocortisone infusion to increase the risk of hyperglycemia and hypoglycemia in critically ill patients.

## Methods

Blood glucose measurements (*n *= 73,400) of patients admitted to the ICU from January 2007 to December 2009 (*n *= 2,167) were analyzed. Logistic regression was used to analyze the effect of hydrocortisone infusion on mild (blood glucose level ≥150 mg/dl) and severe hyperglycemia (≥180 mg/dl) and mild hypoglycemia (≤70 mg/dl) separately. To adjust for severity of disease, patients were stratified in APACHE II score groups (<15; 15 to 24; >24).

## Results

Hydrocortisone infusion was independently associated with mild hypoglycemia (APACHE II score <15, OR 2.40, 95% CI 2.01 to 2.85; APACHE II score 15 to 24, OR 1.53, 95% CI 1.44 to 1.62; APACHE II score >24, OR 1.10, 95% CI 1.05 to 1.15) and severe hyperglycemia in all APACHE II groups (APACHE II score <15, OR 3.26, 95% CI 2.59 to 4.10; APACHE II score 15 to 24 OR 1.45, 95% CI 1.33 to 1.68; and APACHE II score >24 OR 1.09, 95% CI 1.02 to 1.17). Hydrocortisone infusion was independently associated with mild hypoglycemia in patients with APACHE II score 15 to 24 (OR 1.74, 95% CI 1.42 to 2.13) and >24 (OR 1.64, 95% CI 1.42 to 1.90), but not in patients with APACHE II score <15 (OR 1.83, 95% CI 0.94 to 3.55).

## Conclusion

Hydrocortisone increases the risk of dysglycemia in critically ill patients. Whether these dysglycemic effects diminish the beneficial effects of hydrocortisone treatment should be investigated in future studies.
